# Cardioprotective Activity of *Pongamia pinnata* in Streptozotocin-Nicotinamide Induced Diabetic Rats

**DOI:** 10.1155/2015/403291

**Published:** 2015-04-14

**Authors:** Sachin L. Badole, Swapnil M. Chaudhari, Ganesh B. Jangam, Amit D. Kandhare, Subhash L. Bodhankar

**Affiliations:** ^1^Department of Pharmacology, PES's Modern College of Pharmacy, Sector 21, Yamuna Nagar, Nigadi, Pune 411 044, India; ^2^Department of Pharmacology, Poona College of Pharmacy, Bharati Vidyapeeth Deemed University, Paud Road, Erandwane, Pune 411 038, India

## Abstract

*Pongamia pinnata* (L.) Pierre has been used in traditional medicine for the treatment for diabetes and metabolic disorder. The aim of this study was to investigate the effect of petroleum ether extract of the stem bark of *P. pinnata* (known as PPSB-PEE) on cardiomyopathy in diabetic rats. Diabetes was induced in overnight fasted Sprague-Dawley rats by using injection of streptozotocin (55 mg/kg, i.p.). Nicotinamide (100 mg/kg, i.p.) was administered 20 min before administration of streptozotocin. Rats were divided into group I: nondiabetic, group II: diabetic control (tween 80, 2%; 10 mL/kg, p.o.) as vehicle, and group III: PPSB-PEE (100 mg/kg, p.o.). The blood glucose level, ECG, hemodynamic parameters, cardiotoxic and antioxidant biomarkers, and histology of heart were carried out after 4 months after STZ with nicotinamide injection. PPSB-PEE treatment improved the electrocardiographic, hemodynamic changes; LV contractile function; biological markers; oxidative stress parameters; and histological changes in STZ induced diabetic rats. PPSB-PEE (100 mg/kg, p.o.) decreased blood glucose level, improved electrocardiographic parameters (QRS, QT, and QTc intervals) and hemodynamic parameters (SBP, DBP, EDP, max *dP*/*dt*, contractility index, and heart rate), controlled levels of cardiac biomarkers (CK-MB, LDH, and AST), and improved oxidative stress (SOD, MDA, and GSH) in diabetic rats. PPSB-PEE is a promising remedy against cardiomyopathy in diabetic rats.

## 1. Introduction

Diabetic cardiomyopathy is defined as the changes induced by diabetes mellitus in cardiac structure/function in the absence of ischemic heart disease, hypertension, or other cardiac pathologies [1]. Diabetes is multifactorial and has been associated with various disorders including obesity, dyslipidemia, thrombosis, infarction, hypertension, endothelial dysfunction, and coronary artery disease [2]. However, diastolic and systolic dysfunctions are mainly altered apart from traditional cardiac risks parameters such as hypertension, atherosclerosis, and dyslipidemia [3]. Diabetic patients have an increased risk of cardiovascular diseases and these are the major cause of death in them [4, 5]. Cardiomyopathy is a prevalent cause of death in patients with diabetes [6]. The world prevalence of diabetes among adults (aged 20–79 years) will be 6.4%, affecting 285 million adults, in 2010 and will increase to 7.7%, affecting 439 million adults, by 2030 [7]. The global prevalence of diabetes mellitus is forecast to reach 300 million by 2025, and over three quarters of the deaths amongst this population will be expected to result from cardiovascular disease [8]. Individuals with diabetes are at a significantly greater risk of developing both micro- and macrovascular disease and have a cardiac mortality equivalent to that in nondiabetic patients with confirmed heart disease [9].

*Pongamia pinnata *(L.) Pierre (Fabaceae) is popularly known as Indian beech in English [10]. In previous study, we reported that petroleum (PPSB-PEE) and alcoholic extract (PPSB-ALE) of the stem bark of* P. pinnata *(L.) showed antihyperglycaemic activity in diabetic mice [11, 12]. Further, we observed that concomitant administration of PPSB-PEE with synthetic oral hypoglycemic drugs produced synergistic effect in diabetic mice [13]. The preliminary phytochemical analysis of PPSB-PEE showed the presence of alkaloids, terpenoids, triterpenes, flavonoids, steroids, and volatile oils [12]. Cycloart-23-ene-3*β*, 25-diol (B2) isolated from the stem bark of* P. pinnata *possesses antidiabetic activity in diabetic animals [14, 15]. Cycloart-23-ene-3*β*, 25-diol improved the abnormalities of diabetic conditions in diabetic mice due to increased glucagon-like peptide 1 (GLP-1) insulin secretion [16] and has a protective effect on vital organs like heart and kidney [17]. The literature review indicated that* P. pinnata *bark was used by traditional practitioners of medicine for treatment of diabetes and its complications. However, more scientific work is necessary to validate the ethnobotanical claim relating to diabetic complications. Hence, our objective of this study was to evaluate cardioprotective activity of PPSB-PEE in diabetic rats.

## 2. Materials and Methods

### 2.1. Collection, Identification, and Authentication of Plant Materials

The fresh bark of* Pongamia pinnata *(L.) Pierre was collected in August-September 2011 from mature tree near Padali village, Parner Taluka, Ahmednagar district, Maharashtra state, India. The bark of* Pongamia pinnata *was identified and authenticated at Department of Botany, Agharkar Research Institute, Pune, India, and voucher specimen was deposited at that institute (*Pongamia pinnata*: voucher specimen sample number AHMA-23892).

### 2.2. Drugs and Chemicals

Streptozotocin (STZ), nicotinamide (NTM), epinephrine hydrochloride, superoxide dismutase (SOD), and malondialdehyde (MDA) were purchased from Sigma Chemical Co., USA. Petroleum ether (60 : 80), diethyl ether (Merck, India), tween 80 (Research-Lab, India), reduced glutathione (GS H), 5,5′-dithiobis-(2-nitrobenzoic acid) (DTNB) (Hi media, India), thiobarbituric acid (TBA), thiopentone sodium injection (Thiosol Sodium) (Neon Laboratories Ltd., India), creatine kinase-MB isoenzyme (CKMB) (Randox Laboratory, UK), LDH (Ecoline, Merck, India), and GOD/POD kit (Accurex, India) were purchased from respective vendors. All chemicals used were of analytical grade.

### 2.3. Preparation of PPSB-PEE

PPSB-PEE was prepared by using previously reported method [12]. Briefly, weighed quantity (500 g) of air-dried powder (Mesh size-16) of the stem bark of* P. pinnata *was macerated with petroleum ether (3000 mL) at room temperature for 7 days with occasional shaking. This process was repeated with same volume of petroleum ether. Macerates were pooled, transferred to previously weighed Petri dish, and evaporated to dryness at room temperature to obtain 1.6% w/w dried extract.

### 2.4. Experimental Animals and Research Protocol Approval

Male Sprague-Dawley (SD) rats (200–250 g) were procured at our institution animal house facility, Pune, India, and housed in an air-conditioned room at a temperature of 25 ± 2°C and relative humidity of 45% to 55% under 12-h light : 12-h dark cycle. The animals had free access to food pellets (Chakan Oil Mills, Pune, India). Water was provided* ad libitum*. The experimental protocol was approved by the Institutional Animal Ethics Committee (IAEC) constituted in accordance with the rules and guidelines of the Committee for the Purpose of Control and Supervision on Experimental Animals (CPCSEA), New Delhi, India (protocol number MCP/IAEC/20/2011).

### 2.5. Preparation of Drugs Solution

The semisolid PPSB-PEE was emulsified with 2% tween 80 in distilled water to prepare the drug solution. Streptozotocin was dissolved in citrate buffer (pH 4.5) and nicotinamide was dissolved in normal physiological saline.

### 2.6. Induction of Diabetes

Diabetes was induced in overnight fasted Sprague-Dawley rats by using streptozotocin injection (55 mg/kg, i.p.). Nicotinamide (100 mg/kg, i.p.) was administered 20 min before administration of streptozotocin [16]. Hyperglycaemia was confirmed after 3 days using the glucose oxidase peroxidase (GOD/POD) method. Glycemic level control of diabetic rats was made with subcutaneous injections of exogenous human NPH insulin (2 units/day of NPH insulin). Those rats that showed blood glucose levels above 300 mg/dL (diabetic) were selected for the study.

### 2.7. Dose Selection of PPSB-PEE

In our previous reported efficacy study, PPSB-PEE (100 mg/kg, p.o.) showed most promising antihyperglycemic effect in diabetic mice [12, 13]. Based on our previous results, we selected dose (100 mg/kg, p.o.) of PPSB-PEE for this study.

### 2.8. Effect of Chronic Administration of PPSB-PEE in Diabetic Rats

Male Sprague-Dawley rats weighing 200–250 g body weight were divided into three groups and each group contained six rats (*n* = 6) as follows: group I: nondiabetic, group II: diabetic control (tween 80, 2%; 10 mL/kg, p.o.) as vehicle, and group III: PPSB-PEE (100 mg/kg, p.o.). Vehicle and PPSB-PEE were administered for 4 months (once a day) at predetermined time (15 days after injection of streptozotocin and nicotinamide). The condition of the animals was monitored daily at the time of dosing as per experimental design. Blood was collected using the retroorbital plexus of each rat under mild ether anesthesia. Blood glucose was determined by using GOD/POD method at end of the study period.

### 2.9. Serum Cardiac Markers

At the end of experimental period, rats were anaesthetized with diethyl ether. Blood was collected by retroorbital puncture and following serum was separated. Serum levels of aspartate aminotransferase (AST), creatine kinase-MB isoenzyme (CK-MB), and lactate dehydrogenase (LDH) enzymes were measured by automated chemistry analyzer (Microlab 300, Merck, USA) using reagent kits.

### 2.10. Electrocardiographic (ECG), Hemodynamic Parameters

The rats were anesthetized with urethane (1.25 g/kg, i.p.). ECG was recorded using 8-channel recording Power Lab System (AD Instruments, LABCHART 6 software, Australia) and QRS interval, QT interval, heart rate, and QTc interval were recorded. Simultaneously, blood pressure was recorded by polyethylene cannula (PE 50) filled with heparinised saline (100 IU/mL) and connected to pressure transducer. The right carotid artery was cannulated; transducer was filled with heparinised saline and connected to pressure transducer for measurement of blood pressure and systolic blood pressure (SBP), diastolic blood pressure (DBP), and mean arterial blood pressure (MABP) were recorded. Further, left ventricular systolic pressure was measured by means of a Millar mikro-tip transducer catheter (Millar instrument, TX, USA) inserted into the left ventricle via the right carotid artery and connected to a bioamplifier and end diastolic pressure (EDP), maximum *dP*/*dt*, contractility index, and minimum *dP*/*dt* were recorded as standardized in our laboratory.

### 2.11. Effect on Enzymatic Biomarkers of Oxidative Stress

The rats were humanely euthanized after recoding all parameters by cervical dislocation method and the heart was removed and divided into two portions. One portion was used for measurement of myocardial endogenous antioxidant enzymes. Heart tissues were isolated from all rats and were cut into small pieces, placed in chilled 0.25 M sucrose solution, and blotted on a filter paper. The tissues were homogenized in 10% chilled Tris hydrochloride buffer (10 mM, pH 7.4) by tissue homogenizer (Remi Motors, India) and centrifuged at 12000 rpm for 15 min 0°C using Eppendorf's 5810-R high speed cooling centrifuge. The endogenous antioxidant enzymes estimated were malondialdehyde, reduced glutathione, and superoxide dismutase. Malondialdehyde content in supernatant of the rat heart was determined by method of Slater and Sawyer [18]. The assay of reduced glutathione was carried out by method of Moron et al. [19]. The superoxide dismutase activity was determined by method of Misra and Fridovich [20]. Protein concentrations were determined using the method of Lowry et al. [21].

### 2.12. Histopathology of Heart by Hematoxylin-Eosin Staining

The isolated tissue was trimmed into small pieces and preserved in 10% formalin for 24 h. Specimens were cut in sections of 3–5 *μ*m in thickness by microtome and stained by hematoxylin-eosin. The specimens were mounted by disterene phthalate xylene (DPX). The photomicrographs of each tissue section were observed using Cell Imaging software for Life Science microscopy (Olympus Soft Imaging Solution GmbH, Munster, Germany).

### 2.13. Statistical Analysis

The data was expressed as mean ± standard error of mean (SEM). One-way analysis of variance (ANOVA) was applied to test the significance of difference between average biochemical and ECG parameters of different groups and multiple comparisons were determined by post hocDunnett's test. The statistical analysis was performed using GraphPad Prism 5.0 software (GraphPad software, San Diego, CA, USA). *p* < 0.05 was considered statistically significant.

## 3. Results

### 3.1. Effect of PPSB-PEE on Blood Glucose Level in Diabetic Cardiomyopathy in Rats

**Figure 1:**
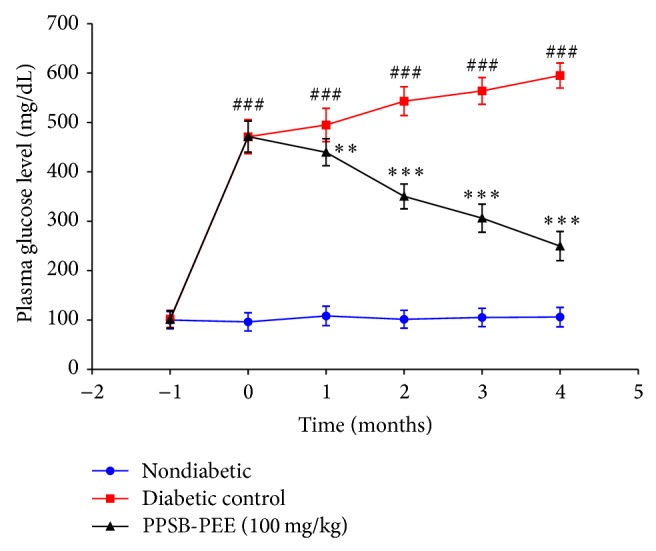
Effect of PPSB-PEE on blood glucose level in rats. Values are mean ± SEM, *n* = 6 in each group; and statistical analysis was carried out by one-way ANOVA followed by post hoc Dunnett's test performed using GraphPad Prism; ^∗∗^ *p* < 0.01; ^∗∗∗^ *p* < 0.001; ns: nonsignificant compared with diabetic group; ^###^ *p* < 0.001; ns: nonsignificant compared with nondiabetic group.

In this study, we observed that diabetic control group was significantly (*p* < 0.001) increased in blood glucose level compared to normal control group. Treatment with PPSB-PEE (100 mg/kg, p.o.) once a day for 4 months at predetermined time caused significant (*p* < 0.001) reduction in blood glucose level compared to diabetic control group. It is thus apparent that PPSB-PEE treatment resulted in better control of blood glucose level in diabetic rats (Figure 1).

### 3.2. Effect of PPSB-PEE on ECG in Rats

ECG recording was found normal in nondiabetic group. QRS interval of diabetic control was significantly (*p* < 0.01) decreased compared to nondiabetic group. After treatment with PPSB-PEE, the QRS interval was significantly (*p* < 0.05) increased compared to diabetic control group. QT and QTc interval of diabetic control showed significant (*p* < 0.001) increase compared to nondiabetic group. QT and QTc intervals showed significant (*p* < 0.05) decrease in PPSB-PEE treatment group compared to diabetic control group. The heart rate of diabetic control showed significant (*p* < 0.01) decrease compared to nondiabetic group. After treatment with PPSB-PEE the heart rate showed significant (*p* < 0.05) increase (Table 1).

### 3.3. Effect of PPSB-PEE on Hemodynamic and LV Function in Rats

Diabetic control group showed significant (*p* < 0.001) decrease in SBP, DBP, EDP, and MABP compared to nondiabetic group. A significant (*p* < 0.001) increase was found in SBP, DBP, and EBP in PPSB-PEE treated diabetic groups. However, nonsignificant fluctuation was observed in MABP after PPSB-PEE treatment (Table 1). Diabetic group showed significant decrease in max *dP*/*dt* (*p* < 0.01), contractility index (*p* < 0.001), and min *dP*/*dt* (*p* < 0.05) compared to nondiabetic group while PPSB-PEE treatment group showed significant (*p* < 0.05) increase in max *dP*/*dt* and contractility index (*p* < 0.001) compared to diabetic control group (Table 1).

### 3.4. Effect of PPSB-PEE on Serum Cardiac Markers in Rats

Serum AST, LDH, and CK-MB level were significantly increased (*p* < 0.001) in diabetic group compared to nondiabetic group. PPSB-PEE treatment significantly reduced serum CK-MB (*p* < 0.001), LDH (*p* < 0.01), and AST (*p* < 0.05) level compared to diabetic group (Table 2).

### 3.5. Effect of PPSB-PEE on Oxidative Stress Markers in Rats

**Figure 2:**
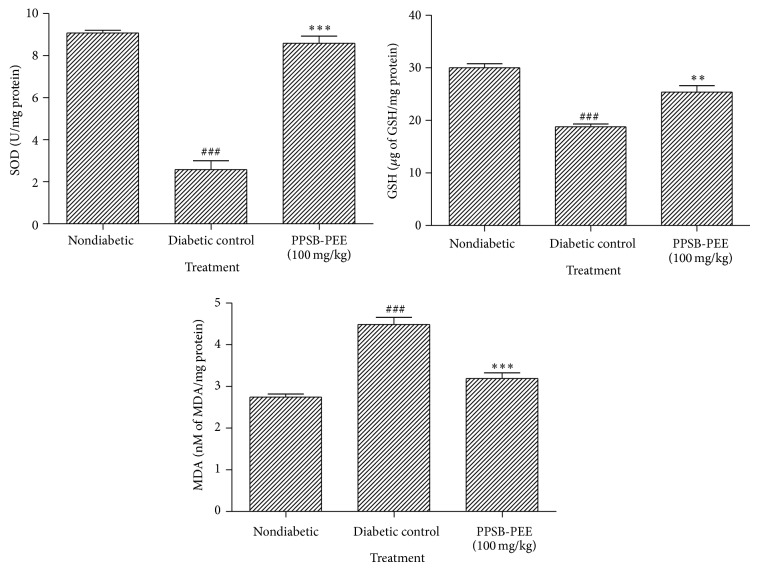
Effect of PPSB-PEE on oxidative stress markers in rats. Values are mean ± SEM, *n* = 6 in each group; and statistical analysis was carried out by one-way ANOVA followed by post hocDunnett's test performed using GraphPad Prism; ^∗∗^ *p* < 0.01, ^∗∗∗^ *p* < 0.001 compared with diabetic control group; ^###^ *p* < 0.001 compared with nondiabetic group.

The SOD and GSH levels were significantly reduced (*p* < 0.001) in the diabetic compared to nondiabetic group. PPSB-PEE treated group showed significantly higher levels of SOD (*p* < 0.001) and GSH (*p* < 0.01) compared to diabetic group. However, MDA level in diabetic group significantly (*p* < 0.001) increased compared to nondiabetic group. Treatment of PPSB-PEE showed significant decrease (*p* < 0.001) compared to diabetic group (Figure 2).

### 3.6. Effect of PPSB-PEE on Histopathology of Heart in Rats

**Figure 3:**
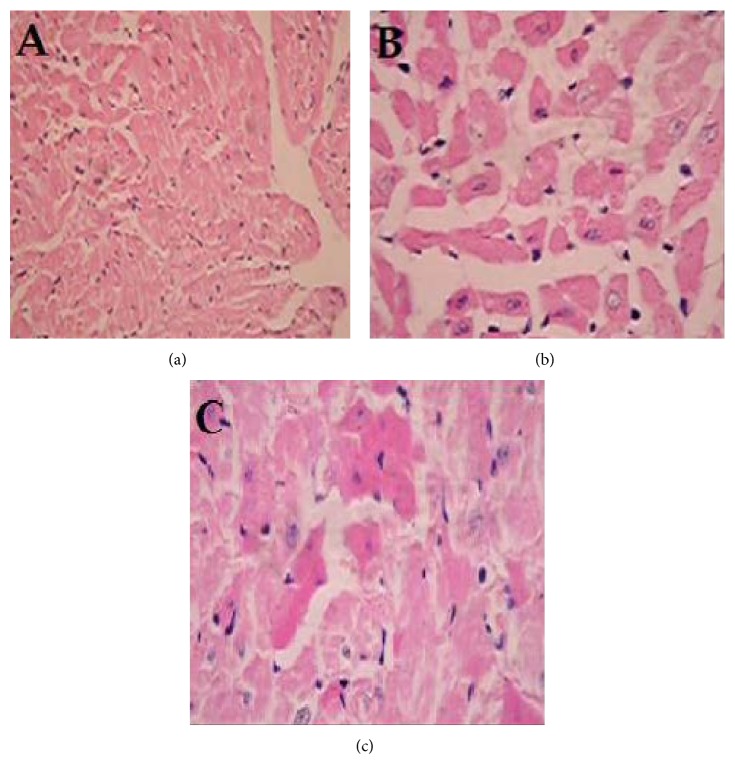
Effect of PPSB-PEE on histopathology of heart in rats. A-group I: nondiabetic, B-group II: diabetic control (tween 80, 2%; 10 mL/kg, p.o.), and C-group III: PPSB-PEE (100 mg/kg, p.o.). (a) Nondiabetic rat showing normal architecture of heart (Grade − −). (b) Diabetic control rat heart showing massive necrosis of heart muscle fibers along with focal mass and fragmentation (+++). (c) PPSB-PEE (100 mg/kg, p.o.) treated rat heart showing minimum pathological changes, that is, swelling of myocardial fibers and focal degeneration (++). Grade −: no injury; Grade ++++: severe injury; Grade ++: moderate injury (magnification 10x).

Hematoxylin-eosin staining of nondiabetic rat heart showed normal architecture of heart (Figure 3(a)). The photomicrographs of diabetic control rat heart showed massive necrosis of heart muscle fibers along with focal mass and fragmentation. Disorganized arrangement with no well-defined boundaries, vascular congestion, cytoplasmic vacuoles, and cytoplasmic eosinophilia were observed severely in myocardial cells (Figure 3(b)). Treatment with PPSB-PEE showed lesser loss of myocardial fibers, myocardial hypertrophy, and cytoplasmic eosinophilia, causing pink coloration of fibers indicating myocardial injury as compared to diabetic control group indicating moderate protection with respect to these parameters (Figure 3(c)).

## 4. Discussion

Diabetic cardiomyopathy is characterized by an early diastolic and later systolic dysfunction with intracellular retention of calcium and sodium and loss of potassium. Cardiac dysfunction occurs at 6 to 12 weeks and even early at 2 weeks from onset of diabetes [22]. In this work, PPSB-PEE (100 mg/kg, p.o.) treatment significantly reduced blood glucose levels in streptozotocin-nicotinamide induced diabetes in Sprague-Dawley rats. PPSB-PEE reduced blood glucose levels due to its ability to stimulate the secretion of insulin from the surviving pancreatic *β* cells. The preliminary phytochemical analysis of PPSB-PEE showed the presence of alkaloids, terpenoids, triterpenes, flavonoids, steroids, and volatile oils. But flavonoids are responsible for stimulating insulin from the surviving pancreatic *β* cells in diabetic mice [12].

Autonomic neuropathy is common complication of both type 1 and type 2 diabetes mellitus. QT interval prolongation is important manifestation of diabetic neuropathy and has been used to screen diabetic patients at risk for sudden cardiac death [23]. Experimental studies with animal models demonstrated that the prolonged action potential resulted from the decreased outward repolarizing potassium currents, especially transient outward potassium currents [24]. However, the exact mechanism of action is not clear yet. The electrocardiogram showed that the QT period was extended in diabetic cardiomyopathy. QTc represents corrected QT interval [22, 23]. In this study, we observed that ECG of diabetic control shows prolonged QT interval and QTc interval compared with nondiabetic group. Moreover, PPSB-PEE (100 mg/kg) protected the cardiac function by reducing the QT interval and QTc interval compared with diabetic control. QRS complex reflects the rapid depolarization of the right and left ventricles [23]. In this study, QRS complex was decreased in diabetic control compared to nondiabetic and PPSB-PEE (100 mg/kg) significantly improved this condition. *P* wave inversion was observed in diabetic group which is indicative of abnormality of SA node. PPSB-PEE treated group did not show inversion of *P* wave.

STZ induced diabetes animals produced systolic and diastolic dysfunction [25]. Diabetic control showed reduced systolic blood pressure, diastolic blood pressure, end diastolic blood pressure, and mean arterial blood pressure. PPSB-PEE (100 mg/kg) treated animals showed significant increase in systolic blood pressure, diastolic blood pressure, and end diastolic blood pressure. The max *dP*/*dt* and min *dP*/*dt* are the maximum rates of pressure development during contraction and relaxation [26]. As previously reported,* in vivo *LV cannulation showed decreased LV max *dP*/*dt* and min *dP*/*dt* after 15 days of STZ injection [27] and after 12 weeks of STZ injection in rats [28]. In this study, diabetic rats show depression in the rate of relaxation (min *dP*/*dt*) and rate of contraction (max *dP*/*dt*) relative to PPSB-PEE (100 mg/kg) treated rats. This result suggests that PPSB-PEE provides sufficient contractile reserve to ameliorate the detrimental effects of diabetes on cardiac contractility. Overall, PPSB-PEE significantly improves contractility index.

Diagnosis of cardiac enzymes is prerequisite in case of diabetic cardiomyopathy. Aspartate aminotransferase (AST), creatine kinase-isoenzyme (CK-MB), and lactate dehydrogenase (LDH) are commonly used as biomarkers for myocardial infarction. Serum creatine phosphokinase activity is a more sensitive indicator in early stage of myocardial ischemia, while peak rise in LDH is roughly proportional to the extent of injury to the myocardial tissue. Also, the integrity of the cardiac apparatus in drug biotransformation and metabolism could be assessed by evaluating the levels of AST, CK-MB, and LDH in serum. These enzymes are tightly bound to the contractile apparatus of the cardiac muscle tissue and any serious insult to the heart muscle will evoke the release of these enzymes into the serum. Diabetic animals show increased levels of CK-MB and LDH in serum [29]. In this study, PPSB-PEE (100 mg/kg) significantly reduced levels of cardiac enzymes such as CK-MB, LDH, and AST compared with diabetic control indicating prevention of cardiac damage and offering cardioprotection.

Hyperglycemia results in the production of reactive oxygen and nitrogen species, which leads to oxidative myocardial injury. Oxidative stress, induced by reactive oxygen and nitrogen species derived from hyperglycemia, causes abnormal gene expression, altered signal transduction, and the activation of pathways leading to programmed myocardial cell deaths. The resulting myocardial cell loss thus plays a critical role in the development of diabetic cardiomyopathy [30, 31]. In this respect, superoxide dismutase is considered a primary enzyme since it is involved in the direct elimination of reactive oxygen species. Superoxide dismutase is an important defense enzyme which catalyzes the dismutation of superoxide radicals [32]. Superoxide dismutase level decreased in diabetes [15, 33]. Reactive oxygen species degrade polyunsaturated lipids forming malondialdehyde. This compound is a reactive aldehyde and is one of the many reactive electrophile species that cause toxic stress in cells and form covalent protein adducts referred to as advanced lipoxidation end-products (ALE), in analogy to advanced glycation end-products (AGE). The production of this aldehyde is used as a biomarker to measure the level of oxidative stress in an organism [34, 35]. Diabetes leads to increase in levels of malondialdehyde [15]. Glutathione, a tripeptide present in millimolar concentrations in all the cells, is an important antioxidant. Reduced glutathione normally plays the role of an intracellular radical scavenger and is the substrate of many xenobiotic elimination reactions [32]. Diabetes leads to decrease in levels of reduced glutathione [15, 36]. In this study, we observed increase in malondialdehyde; decrease in reduced glutathione and superoxide dismutase in heart tissue indicated an increase in oxidative stress in diabetic control. PPSB-PEE was found to possess antioxidant activity. PPSB-PEE (100 mg/kg) significantly reduced oxidative stress by increasing superoxide dismutase, reducing glutathione levels, and decreasing malondialdehyde levels compared with diabetic control.

Histopathological studies reported that mild endomyocardial necrosis was present at 8 weeks of diabetes and a severe focal endomyocardial necrosis was found after 12 weeks of diabetes [22, 37]. In this study, histological changes in diabetic heart showed massive necrosis of heart muscle fibres along with focal mass and fragmentation as compared with nondiabetic heart whereas PPSB-PEE (100 mg/kg) treated diabetic group showed minimal pathological changes; that is, swelling of myocardial fibers and focal degeneration may be due to reduction in oxidative stress and hyperglycemia. PPSB-PEE offered protection to the heart at cellular level.

In conclusion, PPSB-PEE (100 mg/kg, p.o.) decreased blood glucose level, improved electrocardiographic parameters (QRS, QT, and QTc intervals) and hemodynamic parameters (SBP, DBP, EDP, max *dP*/*dt*, contractility index, and heart rate), controlled levels of cardiac biomarkers (CK-MB, LDH, and AST), and improved oxidative stress (SOD, MDA, and GSH) in diabetic rats. Therefore, PPSB-PEE is a promising remedy against diabetic cardiomyopathy. There is open avenue for further cellular and molecular research of the PPSB-PEE.

## Figures and Tables

**Table 1:** Effect of PPSB-PEE treatment on electrocardiographic, hemodynamic changes and LV contractile function in rats.

	Parameters	Nondiabetic	Diabetic control	PPSB-PEE (100 mg/kg)
ECG	Heart rate (BPM)	353.48 ± 15.12	257.95 ± 32.52^##^	338.88 ± 41.08^*^
	QRS interval (ms)	23.12 ± 5.23	13.74 ± 2.03^##^	18.23 ± 3.45^*^
	QT interval (ms)	67.26 ± 1.24	91.00 ± 2.87^###^	73.63 ± 2.02^*^
	QTc interval (ms)	131.08 ± 2.45	194.91 ± 3.88^#^	152.44 ± 0.89^*^

Hemodynamic	SBP (mmHg)	124.31 ± 1.22	87.51 ± 3.54^###^	115.84 ± 1.08^***^
	DBP (mmHg)	91.41 ± 1.44	67.72 ± 1.02^###^	78.65 ± 1.32^***^
	EDP (mmHg)	14.91 ± 2.11	7.13 ± 1.02^###^	18.28 ± 4.27^***^
	MABP (mmHg)	122.25 ± 4.32	79.92 ± 0.12^##^	98.65 ± 0.78

LV function	Max *dP*/*dt* (mmHg/s)	3921.93 ± 322.34	2619.29 ± 80.41^##^	3695.72 ± 287.95^*^
	Min *dP*/*dt* (mmHg/s)	−2513.14 ± 457.65	−1694.52 ± 447.21^#^	−2143.29 ± 398.47
	Contractility index	56.18 ± 5.65	37.75 ± 3.18^###^	43.52 ± 2.98^***^

Data are expressed as mean ± SEM (*n* = 6) and analyzed by one-way ANOVA followed by post hoc Dunnett's test. ^*^ *p* < 0.05;^***^ *p* < 0.001; ns: nonsignificant compared with diabetic group; ^#^ *p* < 0.05; ^##^ *p* < 0.01; ^###^ *p* < 0.001; ns: nonsignificant compared with nondiabetic group.

**Table 2:** Effect of PPSB-PEE treatment on cardiac serum biomarkers in rats.

Parameters	Nondiabetic	Diabetic control	PPSB-PEE (100 mg/kg)
CK-MB (IU/L) concentration	1121.32 ± 101.54	2032.15 ± 163.31^###^	1330.14 ± 45.62^***^
LDH (IU/L) concentration	1290.75 ± 96.23	2816.42 ± 113.57^###^	1766.66 ± 98.75^**^
AST (IU/L) concentration	163.41 ± 21.87	531.92 ± 47.41^###^	332.50 ± 23.62^*^

Data are expressed as mean ± SEM (*n* = 6) and analyzed by one-way ANOVA followed by post hoc Dunnett's test. ^*^ *p* < 0.05; ^**^ *p* < 0.01; ^***^ *p* < 0.001; ns: nonsignificant compared with diabetic group; ^###^ *p* < 0.001; ns: nonsignificant compared with nondiabetic group.
